# Organochlorine and organophosphorus pesticide residues in fodder and milk samples along Musi river belt, India

**DOI:** 10.14202/vetworld.2015.545-550

**Published:** 2015-04-29

**Authors:** Korrapati Kotinagu, Nelapati Krishnaiah

**Affiliations:** Department of Veterinary Public Health and Epidemiology, College of Veterinary Science, Sri Venkateswara Veterinary University, Hyderabad, India

**Keywords:** fodder, gas chromatography, milk, Musi river, pesticide residues

## Abstract

**Aim:**

The present study was conducted to find the organochlorine pesticide (OCP) and organophosphorus pesticide (OPP) residues in fodder and milk samples along Musi river belt, India.

**Materials and Methods:**

Fodder and milk samples collected from the six zones of Musi river belt, Hyderabad India were analyzed by gas chromatography with electron capture detector for OCP residues and pulsated flame photometric detector for the presence of OPP residues.

**Results:**

The gas chromatographic analysis of fodder samples of Zone 5 of Musi river showed the residues of dicofol at concentration of 0.07±0.0007 (0.071-0.077). Among organophosphorus compounds, dimetheoate was present in milk samples collected from Zone 6 at a level of 0.13±0.006 (0.111-0.167). The residues of OCPs, OPPs and cyclodies were below the detection limit in the remaining fodder and milk samples collected from Musi river belt in the present study.

**Conclusion:**

The results indicate that the pesticide residues in fodder and milk samples were well below the maximum residue level (MRL) values, whereas dicofol in fodder and dimethoate in milk were slightly above the MRL values specified by EU and CODEX.

## Introduction

Nowadays more than 800 different kinds of pesticides are used for control of insects, rodents, fungi and unwanted plants in the process of agricultural production. Although most of them leave the products or degrade in soil, water and atmosphere, these chemicals can be transferred to human via food chain [1]. Furthermore, breeding animals and their accommodation can themselves be sprayed with pesticide solution to prevent pest infestations. Consequently, both these contamination routes can lead to bioaccumulation of persistent pesticides in food products of animal origin such as meat, fat, fish eggs and milk [2,3]. Moreover, health departments also used these chemicals for controlling various insects having vector role in spreading the disease like malaria, dengue fever and plague [4,5]. Many pesticides and their residues have been reported as contributory factors in several diseases such as heart disease, cancers, Alzheimer’s disease and parkinsonism [6,7]. Pesticide residues in feed and fodders may transfer into herbivores through food chain of the animals [8]. Due to the lipophilic nature of these pesticides, milk and other fat-rich substances are the key items for their accumulation. These toxicants get into the human body through the food chain and cause serious health problems [7].

Musi river is located on the Deccan platue in the state of Andhra Pradesh, India. However, now the water is highly polluted as 600 million liters per day of untreated sewage water is discharged into Musi river, additionally 14 industrial estates drain their untreated effluents into this river. The agricultural drained water is another source of pollution and this river water is rich in heavy metals, pesticide residues, phenols, oils, grease, alkalis and acids [9]. The self-purifying property of river water is unable to clear the pollution, and the polluted water poses a serious risk to public health especially in areas where river water is used for irrigation.

Keeping this in view of the Musi river pollution and its direct or indirect effect on environment, animal and human system, a study was conducted to analyze the fodder and milk samples on the banks of river Musi for the presence of pesticide residues. The study has been conducted on river Musi, Located in Andhra Pradesh, India.

## Materials and Methods

### Ethical approval

No animals were harmed or given stress during the collection of milk samples.

### Collection of samples

This study was based on 48 fodder and 48 milk samples collected from six divided zones (8 from each zone) (Table-1) on the downstream of Musi river belt, Andhra Pradesh, India in 2013. Zones were divided based on earlier reports on Musi river pollution by Pullaiah [9]. Approximately, 250 g of fodder samples were collected in sterilized polyethylene packs, packed and transported to lab. Sterilized glass bottles were used to collect 250 ml milk samples, labeled and transported to lab in ice pack, they were kept at 4°C until analysis. Samples were subjected to analysis within 24 h from their arrival.

**Table-1:** Selected zones and covered areas along the Musi river belt, Telangana, India.

Zone	Areas covered along Musi river belt
1	Attapur, Langer House, Upper pally, Kishan Bagh, Bahadurpura, Puranapool, Budvel, High court.
2	Chadhar ghat, Malakpet, Morarambagh, Golnaka, Amberpet, Ramanthapur, Nagole, Uppal.
3	Peerzadiguda, K. singaram, Thimaiguda, Pratapa singaram, Korremulla, Bacharam, Bandaraviral, Chinna raviralla.
4	Pillai Palli, Rudravelly, Brahmanapally, Venkiryala, Edulabad, Nadama Khada, Shivareddy gudem, Alinagar.
5	Indriyala, D.R.palli, Wankamamidi, Shaligowraram, Dharmaram, Chittur, Jajireddygudem, Manimadde.
6	Musi reservoir, Yendlapally, Kasarabad, Beemavaram, Dasaphad, M.gudem, Irkigudem, Wazirabad.

### Pesticides analyzed

The residues of certain pesticides of organochlorine *viz*., dichlorodiphenyltrichloroethane (DDT) (o,p’-dichlorodiphenyldichloroethylene [DDE], o,p’-dichlorodiphenyldichloroethane [DDD], p,p’-DDT and o,p’-DDT), dicofol, HCH Isomers (alpha, beta, gamma, delta), cyclodiene compounds (aldrin, endosulfan sulfate and heptachlor) and organophosphates (triazophos, dimetheoate, chlorpyrifos and methyl-chlorpyrifos) in fodder and milk samples collected from six zones of Musi river belt area.

### Chemicals and reagents

Acetonitrile, acetone, dichloromethane, graphitized carbon black, hexane, magnesium sulfate, silica gel, sodium chloride, sodium sulfate, prostate specific antigen (PSA) of high-performance liquid chromatography residue grade obtained from Qualigens and Merck specialties Pvt. Ltd. Analytical standards with >99% purity were obtained from Dr. Ehrenstorfer, Germany during 2012 and stored in deep freeze maintained at −40°C.

### Method validation

The required quantity of (organochlorine and organophosphorus) international standards prepared from certified reference materials were added to each 15 g sample to get fortification levels of 0.05 ppm and 0.1 ppm in three replications each. The AOAC official method 2007.01 with slight modifications was validated for the estimation of the limit of quantification (LOQ) of organochlorine and organophosphorus in fodder and milk. Fodder samples were chopped, and 7.5 g of sample was taken into 50 ml centrifuge tubes and 30 ml of acetonitrile was added and shaken well. The sample was homogenized at 14000-15000 RPM for 2-3 min using heidolp silent crusher then 3 g of sodium chloride was added, mixed well by shaking gently then it was centrifuged at 2500-3000 RPM for 3 min to separate the organic layer, approx. 16 ml of organic layer was taken into a test tube and 9 g of anhydrous sodium sulfate was added to remove moisture [1]. Taken about 0.4 g PSA sorbent and 1.2 g anhydrous magnesium sulfate into 15 ml centrifuge tubes. The 8 ml of organic layer extract was transferred into this 15 ml centrifuge tube, capped and vortex for 30 s, then tubes were centrifuged at 2500-3000 RPM for 5 min then 2 ml of extract was transferred into test tubes and the solvent (acetonitrile) was evaporated turbovap concentrator for GC analysis. Whereas for milk samples, 5 g of milk was taken into 250 ml beaker and 20 g of silica gel and 20 g of anhydrous sodium sulfate was added. Glass column was prepared with 40 ml of dichloromethane over cotton plug, sample was made into slurry with dichloromethane then this was transfer to column and allowed to stand for 90 min then dichloromethane was eluted dropwise, again the sample column was eluted with a mixture of 150 ml acetone: Dichloromethane (2:1 v/w) and anhydrous sodium sulfate was added to the elute, then concentrated to 2-3 ml, 10-15 ml of hexane was added to the concentrate to remove dichloromethane completely, volume was made with n-hexane. Finally, an aliquot of each extract was transferred to 2 ml injection vials to be ready for the analysis.

A Schimadzu 2010 gas chromatography (GC) equipment with a VF-1MS capillary column and with electron capture detector (ECD) and flame photometric detector. All the chemicals were purchased from M/s. Merck specialties Pvt. Ltd and were pesticide residue grade and all pesticide residue standards were purchased from Dr. Erhenstorfer, Germany during 2012. The gas chromatographic analysis was performed under the following conditions (Table-2). A volume of 1 ml sample was injected into the GC; peaks were identified by comparing their retention times with those of standards under the same injection conditions (Table-3). The peak areas of the various peaks whose retention times coincide with the standards were extracted on their corresponding calibration curves to obtain the concentrations.

**Table-2:** Details of GC operating parameters.

GC	GC-Schimadzu 2010
Column	VF-1 ms capillary column 30 m length, 0.25 mm internal diameter, 0.25 mm film thickness; 1% methyl siloxane
Column oven (°C)	260 (isothermal)
Detectors	ECD FPD
Detector temperature (°C)	280
Injector temperature (°C)	260
Injector status	Front injector type 1177 split/splitless Split ratio: 1:5
Carrier gas	Nitrogen, Iolar II, Purity 9.99%
Carrier gas flow (ml min^−1^)	1 ml/min
Makeup flow (ml min^−1^)	35 ml/min
Total run time (min)	60 min

ECD=Electron capture detector, FPD=Flame photometric detector, GC=Gas chromatography

**Table-3:** Details of retention times of OCPs and OPPs under ECD and PFPD.

Retention time	ECD	PFPD
4,4 DDE	27.171	-
2,4-DDD	28.539	-
4,4 DDT	31.312	-
2,4-DDT	29.081	-
Alpha-HCH	14.434	-
Beta-HCH	18.006	-
Gamma-HCH	16.177	-
Delta-HCH	19.366	-
Aldrin	22.026	-
Endo sulfate	33.090	-
Heptachlor	19.704	-
Dicofol	24.082	-
Triazophos	37.406	37.406
Dimethoate	15.300	15.196
Chlorpyrifos	22.111	22.111
ME-chlorpyrifos	18.925	18.925

DDE=Dichlorodiphenyldichloroethylene, DDD=Dichlorodiphenyldichloroethane, DDT=Dichlorodiphenyltrichloroethane, ECD=Electron capture detector, PFPD=Pulsated flame photometric detector

## Results and Discussion

**Figure-1:**
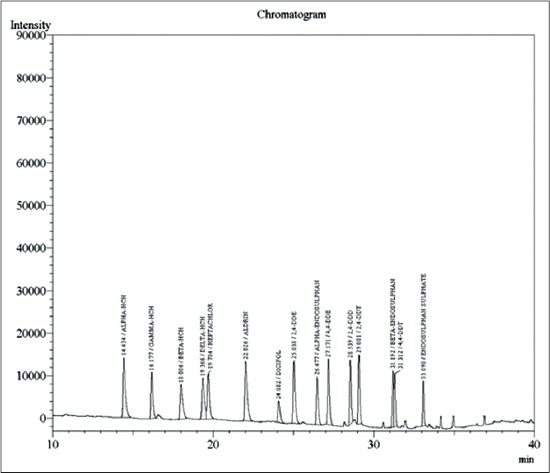
Elution pattern of oranochlorine pesticide standard mixture (0.1 ng).

**Figure-2:**
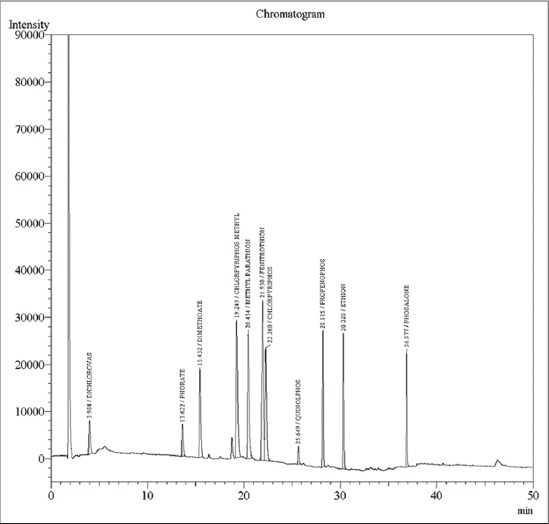
Elution pattern of organophosphorus pesticide standards mixture by electron capture detector.

**Figure-3:**
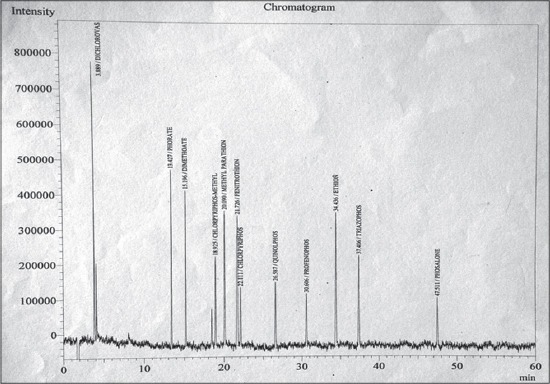
Elution pattern of organophosphorus pesticide standards mixture by pulsated flame photometric detector.

A total of 48 fodder samples and 48 milk samples collected from all the six zones of Musi river belt and were analyzed for OCPs and OPPs residues. Concentration of various residues in each sample was calculated (in mg/kg sample). In the present study, the average recoveries of OCPs in fodder were from 88.05% at 0.05 ppm and 86.71% at 0.1 ppm and in milk were from 88.45% at 0.05 ppm and 91.25% at 0.1 ppm. Average recoveries of OPPs in fodder were 91.27% at 0.05 ppm and 94.67% at 0.1 ppm and in milk were from 91.25% at 0.05 ppm and 86.77% at 0.1 ppm. The efficiency of extraction methodologies were evaluated based on the recoveries of residues, and a recovery of 75-102% is considered as acceptable [10]. Hence, the extraction procedures employed in these experiments were efficient in recovering the maximum amount of residues present in the samples. The elute pattern of various OCPs (0.01 ppm) (Figure-1) and OPPs (0.05 ppm) along with specific retention time are depicted in Figure-2 for ECD and Figure-3 for pulsated flame photometric detector (PFPD). The limit of detection and LOQ for OCPs was 0.01 ppm and 0.05 ppm respectively and for OPPS was 0.05 ppm and 0.05 ppm respectively for both ECD and PFPD.

**Figure-4:**
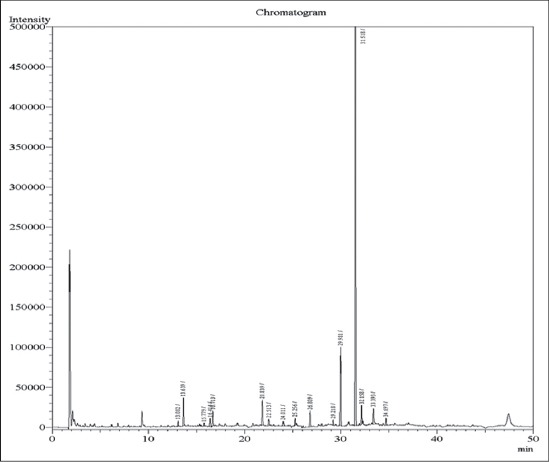
Elution pattern of dicofol from fodder samples of Zone 5 of the Musi river belt.

OCPs compounds p,p’-DDE, o,p’- DDD, p,p’-DDT,o,p’-DDT, Total DDT, dicofol, alpha HCH, beta HCH, gamma HCH, delta HCH, cyclodiene compounds aldrin, endosulphan sulfate, heptachlor and organophosphorus compounds triazophos, methyl chlorpyrifos, chlorpyrifos and dimethoate were analyzed in fodder samples collected from Musi river belt. Fodder samples collected from zone V contain the residual concentration of dicofol of 0.07 ppm (Figure-4) and other organochlorine, organophosphorus and cyclodiene compounds were below detection limit in all other fodder samples in the present study (Table-4).

**Table-4:** Mean residual levels (ppm) of organoclorine and organophosphorus pesticides in fodder and milk samples along Musi river.

Samples	Zones	Total HCH	Total DDT	Dicofol	Aldrin	Endosulphan sulfate	Heptachlor	Triazophos	Methyl chlorpyriphos	Chlorpyriphos	Dimethoate
Fodder	Zone I	BDL	BDL	BDL	BDL	BDL	BDL	BDL	BDL	BDL	BDL
	Zone II	BDL	BDL	BDL	BDL	BDL	BDL	BDL	BDL	BDL	BDL
	Zone III	BDL	BDL	BDL	BDL	BDL	BDL	BDL	BDL	BDL	BDL
	Zone IV	BDL	BDL	BDL	BDL	BDL	BDL	BDL	BDL	BDL	BDL
	Zone V	BDL	BDL	0.07	BDL	BDL	BDL	BDL	BDL	BDL	BDL
	Zone VI	BDL	BDL	BDL	BDL	BDL	BDL	BDL	BDL	BDL	BDL
Milk	Zone I	BDL	BDL	BDL	BDL	BDL	BDL	BDL	BDL	BDL	BDL
	Zone II	BDL	BDL	BDL	BDL	BDL	BDL	BDL	BDL	BDL	BDL
	Zone III	BDL	BDL	BDL	BDL	BDL	BDL	BDL	BDL	BDL	BDL
	Zone IV	BDL	BDL	BDL	BDL	BDL	BDL	BDL	BDL	BDL	BDL
	Zone V	BDL	BDL	BDL	BDL	BDL	BDL	BDL	BDL	BDL	BDL
	Zone VI	BDL	BDL	BDL	BDL	BDL	BDL	BDL	BDL	BDL	0.13

(Each value is mean of 8 replications), Zone I=Attapur, Langer House, Upper pally, Kishan Bagh, Bahadurpura, Puranapool, Budvel, High court, Zone II=Chadhar ghat, Malakpet, Morarambagh, Golnaka, Amberpet, Ramanthapur, Nagole, Uppal, Zone III=Peerzadiguda, K. singaram, Thimaiguda, Pratapa singaram, Korremulla, Bacharam, Bandaraviral, Chinna raviralla, Zone IV=Pillai Palli, Rudravelly, Brahmanapally, Venkiryala, Edulabad, Nadama Khada, Shivareddy gudem, Alinagar, Zone V=Indriyala, D.R.palli, Wankamamidi, Shaligowraram, Dharmaram, Chittur, Jajireddygudem, Manimadde, Zone VI=Musi reservoir, Yendlapally, Kasarabad, Beemavaram, Dasaphad, M.gudem, Irkigudem, Wazirabad, BDL=Below determination level (<0.01)

The prevalence of residues of p,p’ DDE, p,p’ DDT and total DDT in fodder was 5%, 60% and 3% respectively, was reported by Nagra [11]. Residue levels of o,p’ DDT of 0.006 ppm in fodder was reported by Panseri [12]. A residue levels of total DDT of 0.17 ppm in fodder was reported by Nagra [11].

α-HCH and β-HCH residue levels of 0.002 ppm and 0.003 ppm respectively in fodder was reported by Panseri [12].

Residue levels of aldrin of 0.004 ppm and 0.03 ppm in fodder were reported by Panseri [12] and Nagra [11] respectively. A residue level of 0.007 ppm and 0.045 ppm was reported by Panseri [12] and Deka [13] in fodder samples, which is far below the specified MRL value by EU (CE: 698: 2005) is 0.1 ppm, whereas higher levels (0.42 ppm) was reported by Aulakh [14] for endosulphan sulfate. Residual concentration of 0.001 ppm and 0.02 ppm were reported by Panseri [12] and Aulakh [14] respectively in the fodder samples, which are far below the MRL value (0.01 ppm) specified by EU (CE:398: 2005) in fodder for heptachlor. Fagnani *et al*. [15] reported the residual concentration of dimethoate in fodder sample as 0.01 µgl^−1^.

**Figure-5:**
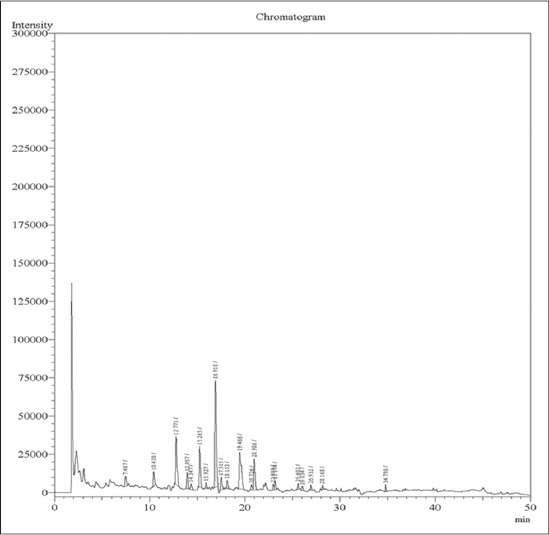
Elution pattern of organophosphorus pesticide residues in milk samples from zone 6 of the Musi river belt.

For milk samples also the same OCP compounds, cyclodiene compounds and organophosphorus compounds conducted for fodder samples were analyzed. Milk samples from zone V contain the residual concentration of dimithioate of 0.13 ppm (Figure-5), which is higher than concentrations of 0.01 µgl^−1^ for dimithioate reported by Fagnani *et al*. [15]. In the present study except dimithioate all other OCPs compounds, cyclodiene compounds and organophosphorus compounds were below the detection limit in milk samples.

The levels of PP DDE were 0.028 ppm in cow milk [16] and 0.039 ppm in mixed milk [17]. Donia [16] reported 0.014 ppm and 0.009 ppm levels of o,p’ DDD in buffalo and cow milk respectively. These findings are far below than the MRL value of 1.25 ppm for milk specified by PFA [18]. The residues of o,p’ DDD of 0.002 ppm was reported by Panseri [12], which is far below than the MRL level (0.05 ppm) specified by EU (CE: 698: 2005). Residue levels of P’P’- DDT of 0.038 and 0.033 ppm were reported by Mohd Aslam [19] and Kampire *et al*. [20] respectively.

Donia [16] reported 0.022 ppm and 0.032 ppm in buffalo and cow milk respectively, whereas Waliszewski *et al*. [21] reported residue levels of 0.0372 and 0.078 in mixed milk for p,p’ DDT. Residue levels of 0.007 ppm and 0.056 ppm in milk were reported by Cerkvenik [22] and Radzyminska [23] respectively, which were below than the MRL value (0.04 ppm) of EU (CE:698: 2005) for o,p’ DDT.

Residue levels of α-HCH of 0.003 ppm, 0.008 ppm and 0.013 ppm were reported by Ahmed and Zaki [24], Cerkvenik [22] and Pardio [17] respectively, which were below than the MRL value (1.25 ppm) of EU (CE: 698: 2005). The β-HCH prevalence of 16.67% and a level of 0.003 ppm were reported by Ahmed and Zaki [24].

Residue levels of aldrin at a level of 0.004 ppm were reported by Ahmed and Zaki [24] in mixed milk, whereas 0.066 ppm and 0.036 ppm were reported by Donia [16] in buffalo milk and cow milk respectively. All the values reported by the above scientists were below than the MRL value (0.2 ppm) of EU (CE: 698: 2005). For endosulphan sulfate, the residue levels of 0.002 ppm and 0.26 ppm were reported by Ahmed and Zaki [24] and Muhammad [25] respectively and prevalence of 9.09% was reported by Ahmed and Zaki [24] in mixed milk sample. The residue levels of heptachlor of 0.003 ppm and 0.022 ppm were reported by Cerkvenik [22] and Donia *et al*. [16] respectively, A prevalence of 16.16% of heptachlor was reported by Ahmad and Zaki [24] in mixed milk.

## Conclusion

From this study, it can be concluded that all the pesticide residues in fodder and milk samples were below the MRL except dicofol in fodder and dimethoate in milk were slightly above the MRL values specified by EU and CODEX it might be due to use of these pesticides on vegetable crops grown on the banks of Musi river belt. However, the results of OPPs in different samples were detected by ECD and confirmed by PFPD, whereas O.C.’s were detected by only ECD but not confirmed by Mass Spectrometry due to non-availability of equipment. Owing to effects on human, animal and environmental health of pesticide residues need for education and awareness among farmers about extensive use of pesticide was envisaged.

## Authors’ Contributions

Both authors have designed the plan of work. KK carried out the sample collection, laboratory work and analyzed the results. KK and KN drafted and revised the manuscript. Both authors read and approved the final manuscript.
